# Digital Microdroplet Ejection Technology-Based Heterogeneous Objects Prototyping

**DOI:** 10.1155/2016/5057347

**Published:** 2016-02-15

**Authors:** Na Li, Jiquan Yang, Chunmei Feng, Jianfei Yang, Liya Zhu, Aiqing Guo

**Affiliations:** Jiangsu Key Laboratory of 3D Printing Equipment and Manufacturing, Nanjing Normal University, Nanjing 210042, China

## Abstract

An integrate fabrication framework is presented to build heterogeneous objects (HEO) using digital microdroplets injecting technology and rapid prototyping. The heterogeneous materials part design and manufacturing method in structure and material was used to change the traditional process. The net node method was used for digital modeling that can configure multimaterials in time. The relationship of material, color, and jetting nozzle was built. The main important contributions are to combine the structure, material, and visualization in one process and give the digital model for manufacture. From the given model, it is concluded that the method is effective for HEO. Using microdroplet rapid prototyping and the model given in the paper HEO could be gotten basically. The model could be used in 3D biomanufacturing.

## 1. Introduction

The structure of biological body is heterogeneous, such as bone, which is of different density. It means the biological products should be made in heterogeneousness for function realization. Nonhomogeneous materials were used for design and manufacture to meet the functional requirements, and they contain a variety of materials [1]. Heterogeneous objects break the limit of traditional parts that contain single material. Design theory and manufacturing method of traditional homogeneous objects cannot be applied directly. Exploring the design theory and manufacturing method of the heterogeneous objects is the current study hot spots. Traditional methods and molding method are based on rapid prototyping (RP). Traditional manufacturing method is based on the functionally graded materials, mainly including vapor deposition method, plasma spraying method, self-propagating high-temperature synthesis method, powder metallurgy, laser cladding method, and centrifugal casting method. These traditional manufacturing methods for functionally graded parts have some disadvantages as in the following sides: it is not possible to manufacture three-dimensional structure precisely, and the bond strength between the gradient layer and the substrate is low which is easy to crack, and material distribution cannot be controled accurately [2]. Yakovleva et al. study the laser direct forming method for three-dimensional objects with gradient material [3]. Lappo et al. developed multimaterial selective laser sintering (SLS) equipment which is based on the discrete molded, used to make continuous multimaterial prototypes [4]. Cho et al. reported the molding equipment developed based on the three-dimensional printing (3DP) process proposed by the MIT, and the device uses multiple digital print nozzles injection molding material to create a three-dimensional model [5]. Yang and Evans developed the SLS process based multimaterial powder spray equipment used to produce three-dimensional functionally gradient material component [6]. Bremnan et al. developed multimaterial layered manufacturing equipment that can be commercialized and used to process electric ceramic pieces [7]. Choi and Cheung research on multimaterial laminate manufacturing process based topology level path planning [8]. Li et al. have a research on geometry and material information description of CAD model for ideal material parts, slicing the algorithm of CAD model [9]. They developed a molding system prototype of the ideal material; the system uses screw extrusion continuous jet and the nozzle squeeze, the molten ABS silk to produce multifunctional material components. Zhang et al. studied multibranch, multilayer structure and the artificial bone bracket containing heterogeneous porous structure with gradient function of the bioengineering organization [10]. Zhizhong et al. study the forming process of complex shapes silicon carbide ceramic components based on stereolithography technology manufacturing [11–15].

Some molding material was applied in these molding methods which is very limited, and some lower molding precision and the molding efficiency are low and formed certain limitations for controlling the multiphase material in the space within heterogeneous objects precisely. Although this molding method or system is not yet mature or perfect, it gave a basis for the rapid manufacturing of heterogeneous objects.

The RP-based manufacturing method for heterogeneous objects, due to the discrete-accumulation principle, makes simultaneous molding in geometry and material distribution possible and occupies an important position in terms of forming heterogeneous objects in recent years [12, 16, 17]. Domestic and foreign research institutions and departments are committed to the RP molding of heterogeneous objects and have achieved preliminary results [18, 19]. This paper presents a digital microdroplet ejection technology of manufacturing methods for heterogeneous objects and a designed prototyping system. A good and effective heterogeneous material injection shaping models were given for 3D digital manufacturing.

The structure of this paper is as follows: the instruction was given in Section 1. The art of manufacturing was given in Section 2. The CAD model of heterogeneous objects was present in Section 3. The CAD model data processing for heterogeneous objects was given in Section 4. The droplets injection molding of multiphase material parts was given in Section 5. And the conclusion was given in Section 6.

## 2. Design and Manufacture Processes of Heterogeneous Objects

**Figure 1:**
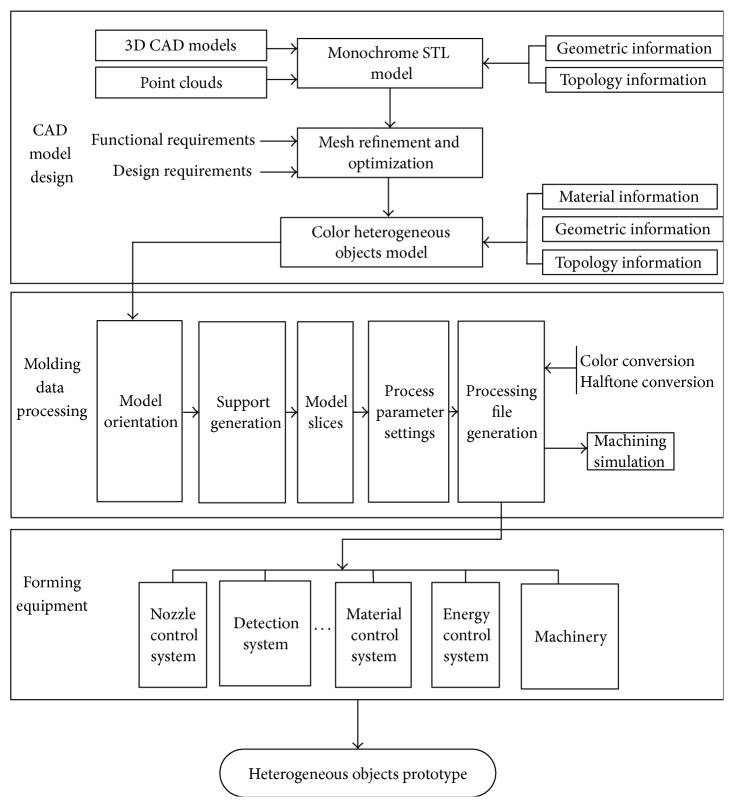
Molding process of HEO prototype system.

Heterogeneous objects, especially the manufacturing of functionally graded materials, must be formed in a single manufacturing, which is difficult for traditional manufacturing method to cope with due to the materials and structures. The characteristics of accurate injecting and accumulation process point-by-point of the 3D printing in rapid prototyping provide a possible means of production for heterogeneous objects and are capable of unifying the preparation of materials and manufacturing of parts. The system structure is in Figure 1.

A molding method for heterogeneous objects combines the designing CAD model [20] of the multimaterial, 3D printing technology, composite materials preparation process, and design and manufacturing process is shown in Figure 1. This process includes three parts: CAD model of heterogeneous objects designing, the molding data processing, and forming equipment. The object was first described in point clouds and transformed into STL file with the points which store the key information of material and structure of object. The slicing codes were translated to print road of the forming equipment. The forming equipment is called three-dimensional printing (3DP) which is used for fabricating prototypes directly from CAD models in a layer-by-layer manner. There was a large variety of 3DP processes introduced in the past decade. Successive layers of prototype are printed using digital model until the whole prototype is fabricated.

## 3. The CAD Model of Heterogeneous Objects

### 3.1. Design Process of Heterogeneous Objects

**Figure 2:**
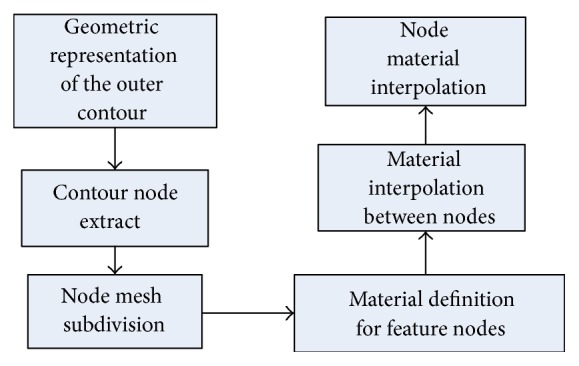
CAD design process models for heterogeneous objects.

CAD model of heterogeneous objects includes structural model and materials model, and materials model design is based on the structure model, and the design process is as shown in Figure 2.

Firstly, from CAD STL model of heterogeneous objects, which aim for the contour surface geometry, the vertex of each STL triangular facets could be gained that is the node data. Then, subdivide the model node grid based on the design requirements and functional requirements of heterogeneous objects. Before the process of assigning the material for nodes, material definition of the feature nodes should be decided, and the subsequent interpolation operation is carried out on the material interpolation between the nodes.

### 3.2. Node Material Assignment

**Figure 3:**
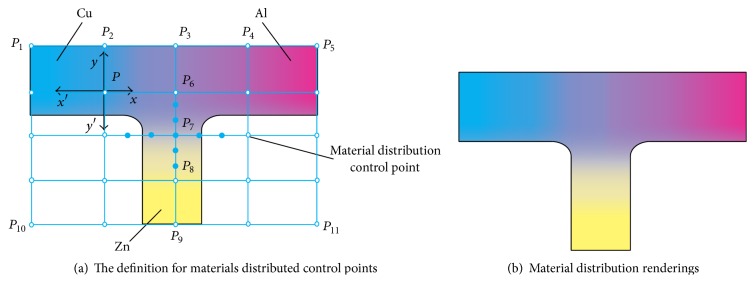
Material distribution definition.

The functionally gradient material plane model is shown in Figure 3, for example, which is composed of three metallic materials Zn, Al, and Cu. The outer feature points *P*_1_, *P*_5_, *P*_11_, *P*_10_ constitute the outline frame and the device feature points *P*_2_, *P*_3_, *P*_4_, *P*_6_, *P*_7_, *P*_8_, and *P*_9_ constitute the subdivision feature nodes and could be appropriately subdivided as in Figure 3(a).

The definition of the feature nodes in the CAD model of the heterogeneous objects is according to (1) and Table 1 for the feature node material assignment

(1) Pi=s1,s2,…,sl,x1,x1′,y1,y1′,…,xj,xj′,yj,yj′,∑l=1ksl=1,sl∈0,1,xt=MPt+1st,xMPtst,x,xt′=MPt−1si,xMPtst,x,yt=MPt+1st,yMPtst,y,yt′=MPt−1st,yMPtst,y,

where *k* is the total number of material species contained in the model. *s*_*l*_ represents the body components of the *l*th material at *P*-node. *l* is the sum of body components of all materials. *x*_*t*_ and *x*_*t*_′ are the distribution vectors of *t*th material at the two opposite directions along the *x*-axis, respectively, indicating the changes trend of the material. The value is the ratio of the material body component between the node and next node along *X* direction. The greater the absolute value of the vector, the more intense the material change around this point. The vector is equal to 1 and means the same body component of the node and the next node, 0 means the contour points, −1 means the same material body component with the prior node, +*∞* means that the point does not contain this material, but the neighboring direction contains the material. *y*_*t*_ and *y*_*t*_′ are similar to *x*_*i*_ and *x*_*i*_′ definition.

There are two special cases according to material definition description above. In one case, the neighboring nodes do not contain the material; four material vectors of a node are zero and the material body component is nonzero. In another case, it is called the material mutations area when four material vectors of the node are +*∞* which stands for the material fracture area.

### 3.3. Material Node Subdivision

**Figure 4:**
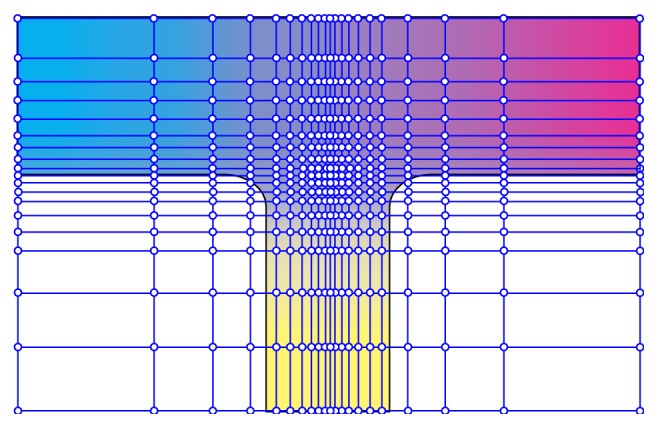
Material definition mesh subdivision.

In order to improve the accuracy of material distribution definition, set further grid refinement as shown in Figure 3(a). The refinement principle is based on the material distribution vector of the original nodes; that is, the material changes the curvature, and the greater the change of the curvature, the higher the subdivision point density, as shown in Figure 4.

The definition of the nodes in Figure 4 was given as in Table 1. The density of nodes was related to the number of materials types. The node density was given as *d*_*n*_ = 2^*n*^ × *d*_0_, where *d*_0_ is the density of a single material.

### 3.4. The Material Interpolation Method between the Nodes

The material distribution among the nodes gradually is designed according to the existing material value of the above-mentioned nodes using the linear interpolation equation (2)

(2) MPjx,αi=1−αixMPix+αixMPi+1x,MPjy,αi=1−αiyMPiy+αiyMPi+1y,αix=dPjx,Pi+1xdPix,Pi+1x,αiy=dPjy,Pi+1ydPiy,Pi+1y,0≤αix≤1,  0≤αiy≤1,

where *P*_*j*_ means the random interpolation points between points *P*_*i*_ and *P*_*i*+1_, *d*(·, ·) denotes the Euclidean distance between any two points in space, and *α*_*i*_(*x*) stands for the material linear interpolation rights of *i*th material *S*_*i*_ along the *X* direction between points *P*_*i*_(*x*) and *P*_*i*+1_(*x*). *α*_*i*_(*y*) stands for the material linear interpolation rights of *i*th material *S*_*i*_ along the *Y* direction between points *P*_*i*_(*y*) and *P*_*i*+1_(*y*).

The gradient distribution of three materials of is shown in Figure 3(b) based on the node assignment and node interpolation method.

## 4. The CAD Model Data Processing for Heterogeneous Objects

After the completion of the material assignment on each node, STL model should be sliced into layers to get the series slices. Each slice is composed of a plurality of contours, and the material distribution of each contour vertex can be obtained by the linear interpolation. Each grid is quadrilateral after refining the mesh within each slice. Material values of each grid node are gotten from interpolation operation according to (2) based on the vertex material values of the outer contour. Subsequently, the material distribution within each grid can also be obtained one by one in order to get the material distribution at any point within each slice.

The color layout of desktop publishing industry has similarities with the material distribution within two-dimensional geometric plane. Two-dimensional cross section of multimaterial model can be seen as a color image, to establish the mappings between color space and materials space.

According to the integration method of design and manufacturing for the HEO, any point *P*_*z*_ can be represented by the expression (3) in the molding process:

(3) $$\begin{array}{c} P_{z}\left(\;k,i,j,m_{l}\;\right), \end{array}$$

where *P*_*z*_ is located in the *k-*layer in *z* direction, *i* and *j* are the *i*th row and *j*th column pixels of geometric information in CMY mode graphics of the slice layer, and *m*_*l*_ ∈ *M* is the material distribution matrix of the point (material information).

**Figure 5:**
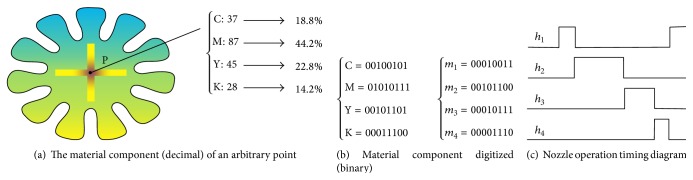
The material component of arbitrary point correspondence relationship with nozzle actions.

*M* matrix in (4) is determined by material information of the point in the modeling process. The example shown in Figure 5 is containing four kinds of material for heterogeneous model. The material components of any point *P* can be obtained through design process in the following sequence: firstly the aforementioned color-material mapping and secondly material assignment for nodes and finally material interpolation between nodes.  Figure 5(a) gives the color component and material proportion occupied by each component of the point *P*, and each point within the heterogeneous entity shall be in accordance with (4), and the sum of the volume of the materials components is 1

(4) $$\begin{array}{c} \overset{4}{\underset{l=1}{\sum }}m_{l}=1. \end{array}$$

To get precisely the material distribution of the model for each point within the heterogeneous entity, the molding process should be precisely on material volume component and be accurately controlled on the material molding. For the digitized droplet injection molding method, the relationship of nozzle and the material is one-to-one, and in order to facilitate the subsequent digital ejection for nozzles the material points are converted in proportion to the corresponding binary value. The body components of *m*_1_, *m*_2_, *m*_3_, and *m*_4_ materials were 18.8%, 44.2%, 22.8%, and 14.2%, as seen in Figure 5(b).

Drop-on-demand method is used for microdroplet ejection system with multinozzle, and the injection control system is shown in Figure 5(b). It shows that material binary value of each point is carried out by high-speed switching operation. After one nozzle completes ejection, the next nozzle begins to eject other materials. The corresponding operation time sequence chart of the nozzles is shown in Figure 5(c) in order to accurately achieve the material stacked for each point within every layer.

Binary manner is used here for every material. What should be noted is that the injection time is an integer multiple of the minimum pulse period. Therefore, there will be error caused by transforming problems inevitably; the injection system expands the material body fraction to 100 times to reduce for the material forming deviation, and rounding method is used to round the number of injection pulses of every material. The injection pulse frequencies of materials at the point *P*_*z*_ were 1878, 4416, 2284, and 1421, respectively, in accordance with Figure 5(c).

## 5. The Droplets Injection Molding of Multiphase Material Parts

Our group proposed and developed a new molding method that is the digital microdroplet ejection technology-based heterogeneous objects directly manufactured based on the research results of multimaterial droplet injection molding and rapid prototyping and the improvement of polymer materials use experience. The main idea of the method is to carry on the concurrent design of the geometry and topology shape (monochrome STL surface model data) based on the color STL model, and the material structure (color information) is according to the functional requirements of parts. Slice the layer of color STL model containing the structural and material information, which obtains a series of colored slices in order to analyze the color information and structural information of each processing unit. New microdroplet ejection technology and rapid prototyping technology are used in molding process. The microdroplet ejection of the concentrated suspension contained material particles and UV photosensitive resin or low melting point alloy melt through a fine nozzle, thereby obtaining the HEO prototypes.

**Figure 6:**
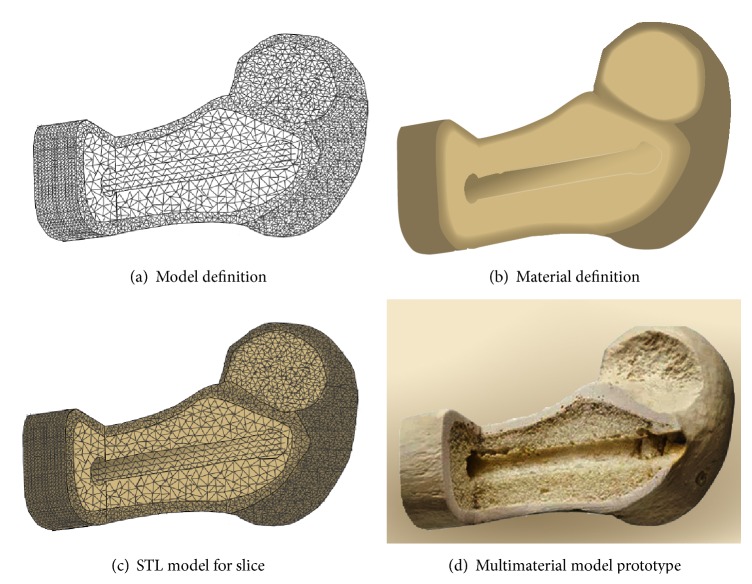
Heterogeneous objects model color prototypes.

Figure 6 used the parallel design and manufacturing method of the heterogeneous objects to produce the irregular hemispherical bone possessing two materials (model data in Table 2). The common STL model only has rough geometric information without material information.  Figure 6(a) is the model of the bone.  Figure 6(b) stands for the foundation for material information description accurately.  Figure 6(c) is the aforementioned refined STL model which gave material information and its renderings.  Figure 6(d) is the prototypes processed by heterogeneous material molding system. The manufacture parameter was gotten as shown in Table 2. Dimensions (mm) were the manufacturing area, volume (mm^3^) is the amount of the material consumed, triangles are the sliced information, and the layer thickness (mm) is the manufacture height in one layer.

The modeling method for heterogeneous objects based on the STL format and space microtetrahedron is proposed adopting STL common data formats in RP areas, and the microtetrahedral mesh refinement is uniformed based on part features. The heterogeneous object was decomposed layer-by-layer, giving material information to grid nodes. And grid was constructed on the surface including internal structure and material information. The method realized parallel design of structure and materials for heterogeneous objects. Such space microtetrahedral-based modeling method of heterogeneous objects integrated the design process of structural design, material design, and model visualization, which has the following advantages compared with other methods.

1. It is easy to dock with the existing CAD design software and RP molding equipment using STL common data format and provided the data protection for CAD and CAM integration of heterogeneous objects.
2. It gave the foundation for fine and fast visualization of heterogeneous objects in CAD models in following the process. During the rendering process, only the color of the STL facets located on the surface of heterogeneous objects needs to be set simplifying the displayed processing problem within the microtetrahedron. It is efficient in color processing time.
3. It is a new way for point cloud in CAD data to reconstruct heterogeneous objects using the grid nodes definition.
4. Facilitate the storage of heterogeneous objects orderly using data in layer-by-layer formal, and it is easy for hierarchical processing and molding of CAD model.

The modeling method of heterogeneous objects is still in the stage of perfection. Future research will focus on the following aspects: dynamic modeling of heterogeneous objects, finite element analysis of CAD models for heterogeneous objects, rapid prototyping methods, and formation mechanism of heterogeneous objects.

## 6. Conclusions

The above-mentioned research has important theoretical and practical significance meanings in several aspects: good for enhancing the integration of digital design and manufacture of heterogeneous objects, useful for a further understanding and revealing a complex physical phenomenon and mechanism generated during the manufacturing process and good to improve the molding quality and other aspects of the heterogeneous objects.

This manufacture method integrated the designing and forming approach including structural design, material design, and model visualization integration using digital droplet injection technology and rapid prototyping technology. It can also combine the polymer materials, low melting point alloy materials, ceramic particles, and other organic and inorganic substances. This method provides a new model for fast and precise manufacture of heterogeneous objects.

## Figures and Tables

**Table 1:** The material distribution definition for nodes.

Nodes	Node material values indicate
*P*_1_	(1,0, 0, 0.75, 0,0, 1, +*∞*, 0,0, −1,0, 0,0, 0)
*P*_2_	(0.75, 0.25, 0,0.67, −1.33, 0, −1,2, 0,0, −1,0, 0,0, 0)
*P*_3_	(0.5,0.5,0, 0.5, −1.5,0, −1,1.5, −0.5, 0, −1,0, 0,0, 0)
*P*_4_	(0.25, 0.75, 0, 0, −2, 0, −1,1.33, −0.67, 0, −1,0, 0,0, 0)
*P*_5_	(0,1, 0,0, −*∞*, 0, −1, 0, −0.75,0, −1,0, 0,0, 0)
*P*_6_	(0.5, 0.5, 0, 0.5, −1.5, 1, −0.67,1.5, −0.5, 1, −0.67,0, 0,0, −*∞*)
*P*_7_	(0.33, 0.33, 0.33, 1, −1,1.5, −0.5,1, −1,1.5, −0.5,1, −1,0, −2)
*P*_8_	(0.16,0.16,0.67,1, −1,2, 0,1, −1,2, 0,1, −1,0.5, −1.5)
*P*_9_	(0,0, 1,0, 0, +*∞*, 0,0, 0, +*∞*, 0,1, −1,0.67,0)

**Table 2:** The HEO model information.

Dimensions (mm)	205.012 × 143.032 × 88.049
Volume (mm^3^)	235410.123
Triangles before Remesh	3550
Triangles after Remesh	36248
Layer thickness (mm)	0.1

## References

[1] Hopkinson N., Hague R., Dickens P. (2006). *Rapid Manufacturing: An Industrial Revolution for the Digital Age*.

[2] Jiquan Y., Guocai X. (2006). *Rapid Prototyping Technology*.

[3] Yakovleva A., Trunovaa E., Greveya D., Pilloz M., Smurov I. (2005). Laser-assisted direct manufacturing of functionally graded 3D objects. *Surface and Coatings Technology*.

[4] Lappo K., Jackson B., Wood K. Discrete multiple material selective laser sintering (M^2^SLS): experimental study of part processing.

[5] Cho W., Sachs E. M., Patrikalakis N. M., Troxel D. E. (2003). A dithering algorithm for local composition control with three-dimensional printing. *Computer Aided Design*.

[6] Yang S., Evans J. R. G. (2004). A multi-component powder dispensing system for three dimensional functional gradients. *Materials Science and Engineering A*.

[7] Bremnan R. E., Turcu S., Hall A. (2003). Fabrication of electroceramic components by layered manufacturing (LM). *Ferro-Electrics*.

[8] Choi S. H., Cheung H. H. (2006). A topological hierarchy-based approach to toolpath planning for multi-material layered manufacturing. *Computer-Aided Design*.

[9] Li R., Rui Y., Dongming G. (2008). Concurrent design of geometry and material for no-homogeneous objects. *Mechanical Engineering of China*.

[10] Zhang R., Yan Y., Lin F. (2008). Low temperature rapid prototyping (LT-RP) and green manufacturing. *Manufacturing Technology & Machine Tool*.

[11] Zhizhong C., Dichen L., Jishun L. (2008). Research on a new technology of complex shape of the silicon carbide ceramic. *Chinese Journal of Mechanical Engineering*.

[12] Toprakci H. A. K., Kalanadhabhatla S. K., Spontak R. J., Ghosh T. K. (2013). Polymer nanocomposites containing carbon nanofibers as soft printable sensors exhibiting strain-reversible piezoresistivity. *Advanced Functional Materials*.

[13] Lan J. (2013). *Design and Fabrication of a Modular Multi-Material 3D Printer*.

[14] Na L., Jiquan Y., Jihong C., Weixing Q. (2014). Digital printing of the thin film sensor with sharp edge based on electrodynamics 3DP. *The Open Electrical & Electronic Engineering Journal*.

[15] Chumnanklang R., Panyathanmaporn T., Sitthiseripratip K., Suwanprateeb J. (2007). 3D printing of hydroxyapatite: effect of binder concentration in pre-coated particle on part strength. *Materials Science and Engineering C*.

[16] Fan Z., Ho J. C., Takahashi T. (2009). Toward the development of printable Nanowire electronics and sensors. *Advanced Materials*.

[17] Lee J.-S., Kim S.-Y., Kim Y.-J. (2008). Design and evaluation of a silicon based multi-nozzle for addressable jetting using a controlled flow rate in electrohydrodynamic jet printing. *Applied Physics Letters*.

[18] Logothetidis S. (2008). Flexible organic electronic devices: materials, process and applications. *Materials Science and Engineering B: Solid-State Materials for Advanced Technology*.

[19] Goghari A. A., Chandra S. (2008). Producing droplets smaller than the nozzle diameter by using a pneumatic drop-on-demand droplet generator. *Experiments in Fluids*.

[20] Zhang Y. D., Wang S. H., Ji G. L., Dong Z. (2015). Exponential wavelet iterative shrinkage thresholding algorithm with random shift for compressed sensing magnetic resonance imaging. *IEEJ Transactions on Electrical and Electronic Engineering*.

